# Determinants of malaria testing at health facilities: the case of Uganda

**DOI:** 10.1186/s12936-021-03992-9

**Published:** 2021-12-04

**Authors:** Ruth N. Kigozi, JohnBaptist Bwanika, Emily Goodwin, Peter Thomas, Patrick Bukoma, Persis Nabyonga, Fred Isabirye, Paul Oboth, Carol Kyozira, Mame Niang, Kassahun Belay, Gloria Sebikaari, James K. Tibenderana, Sam Siduda Gudoi

**Affiliations:** 1 MAPD Project, US President’s Malaria Initiative, Kampala, Uganda; 2 Malaria Branch, Centers for Disease Control and Prevention, US President’s Malaria Initiative, Atlanta, GA 30329 USA; 3 Infectious Disease Institute, Kampala, Uganda; 4 Ministry of Health, Kampala, Uganda; 5 Malaria Branch, Centers for Disease Control and Prevention, US President’s Malaria Initiative, Kampala, Uganda; 6 US President’s Malaria Initiative, US Agency for International Development, Kampala, Uganda; 7 Malaria Consortium, London, UK

**Keywords:** Malaria, Malaria diagnostic practices

## Abstract

**Background:**

The World Health Organization (WHO) recommends prompt malaria diagnosis with either microscopy or malaria rapid diagnostic tests (RDTs) and treatment with an effective anti-malarial, as key interventions to control malaria. However, in sub-Saharan Africa, malaria diagnosis is still often influenced by clinical symptoms, with patients and care providers often interpreting all fevers as malaria. The Ministry of Health in Uganda defines suspected malaria cases as those with a fever. A target of conducting testing for at least 75% of those suspected to have malaria was established by the National Malaria Reduction Strategic Plan 2014–2020.

**Methods:**

This study investigated factors that affect malaria testing at health facilities in Uganda using data collected in March/April 2017 in a cross-sectional survey of health facilities from the 52 districts that are supported by the US President’s Malaria Initiative (PMI). The study assessed health facility capacity to provide quality malaria care and treatment. Data were collected from all 1085 public and private health facilities in the 52 districts. Factors assessed included supportive supervision, availability of malaria management guidelines, laboratory infrastructure, and training health workers in the use of malaria rapid diagnostic test (RDT). Survey data were matched with routinely collected health facility malaria data obtained from the district health information system Version-2 (DHIS2). Associations between testing at least 75% of suspect malaria cases with several factors were examined using multivariate logistic regression.

**Results:**

Key malaria commodities were widely available; 92% and 85% of the health facilities reported availability of RDTs and artemether–lumefantrine, respectively. Overall, 933 (86%) of the facilities tested over 75% of patients suspected to have malaria. Predictors of meeting the testing target were: supervision in the last 6 months (OR: 1.72, 95% CI 1.04–2.85) and a health facility having at least one health worker trained in the use of RDTs (OR: 1.62, 95% CI 1.04–2.55).

**Conclusion:**

The study findings underscore the need for malaria control programmes to provide regular supportive supervision to health facilities and train health workers in the use of RDTs.

## Background

Malaria remains one of the main global health challenges with 3.4 billion people at risk leading to 229 million cases and 409,000 deaths each year, the bulk of which are reported in sub-Saharan Africa [1]. Uganda ranks 5th among the highest contributors of malaria cases in this region. Malaria is endemic in 95% of Uganda, and is responsible for 20% of outpatient visits, 15% of hospital admissions and up to 5–10% of inpatient deaths [2, 3]. The World Health Organization (WHO) recommends prompt and effective diagnosis and treatment as one of the key interventions to control malaria [4]. Diagnosis should be guided by parasitological confirmation with either microscopy or malaria rapid diagnostic tests (RDTs) for all persons of all ages in all epidemiological settings [4]. Microscopic examination of blood smears has been considered the gold standard for malaria diagnosis, but maintaining adequate microscopy standards is challenging in resource limited settings and thus parasite based rapid diagnostic tests have been recommended as having comparable precision and incorporated into clinical guidelines for malaria endemic countries [5]. However, in sub-Saharan Africa, malaria diagnosis is still influenced by clinical symptoms with patients and care providers often attributing all fevers as due to malaria. This practice stems from high malaria endemicity where most fevers are assumed to be malaria, traditional health perceptions, and issues related to laboratory testing including inadequate supplies, low numbers of laboratory staff numbers coupled with limited diagnostic capacity and high backlog, among others [6].

In Uganda, the Ministry of Health and National Malaria Control Division (NMCD) established a policy of test and treat for malaria in 2005. This policy requires diagnostic testing for every suspected malaria case (defined as those presenting with fever) and restricting treatment for malaria to only those cases with evidence of a positive parasitological test result. Implementation of this policy was limited in the first decade of its adoption. Testing among those suspected was at only 24% in 2010, though this rose impressively to 59% in 2013 [7, 8]. Uganda hoped, through its 5-year (2014–2020) malaria reduction strategy, to have at least 75% of those suspected malaria cases being tested in 2019, increasing to 84% in 2021 [8]. To ensure Uganda can reach its test and treat goal, there is a need to understand the factors that influence and can possibly sustain malaria parasitological testing at health facilities. This study was conducted with this purpose in mind and to generate knowledge that will help malaria control programmes ensure that all health facilities are able to implement the recommendation of parasitological testing for all patients suspected to have malaria.

## Methods

### Study design

**Fig 1.**
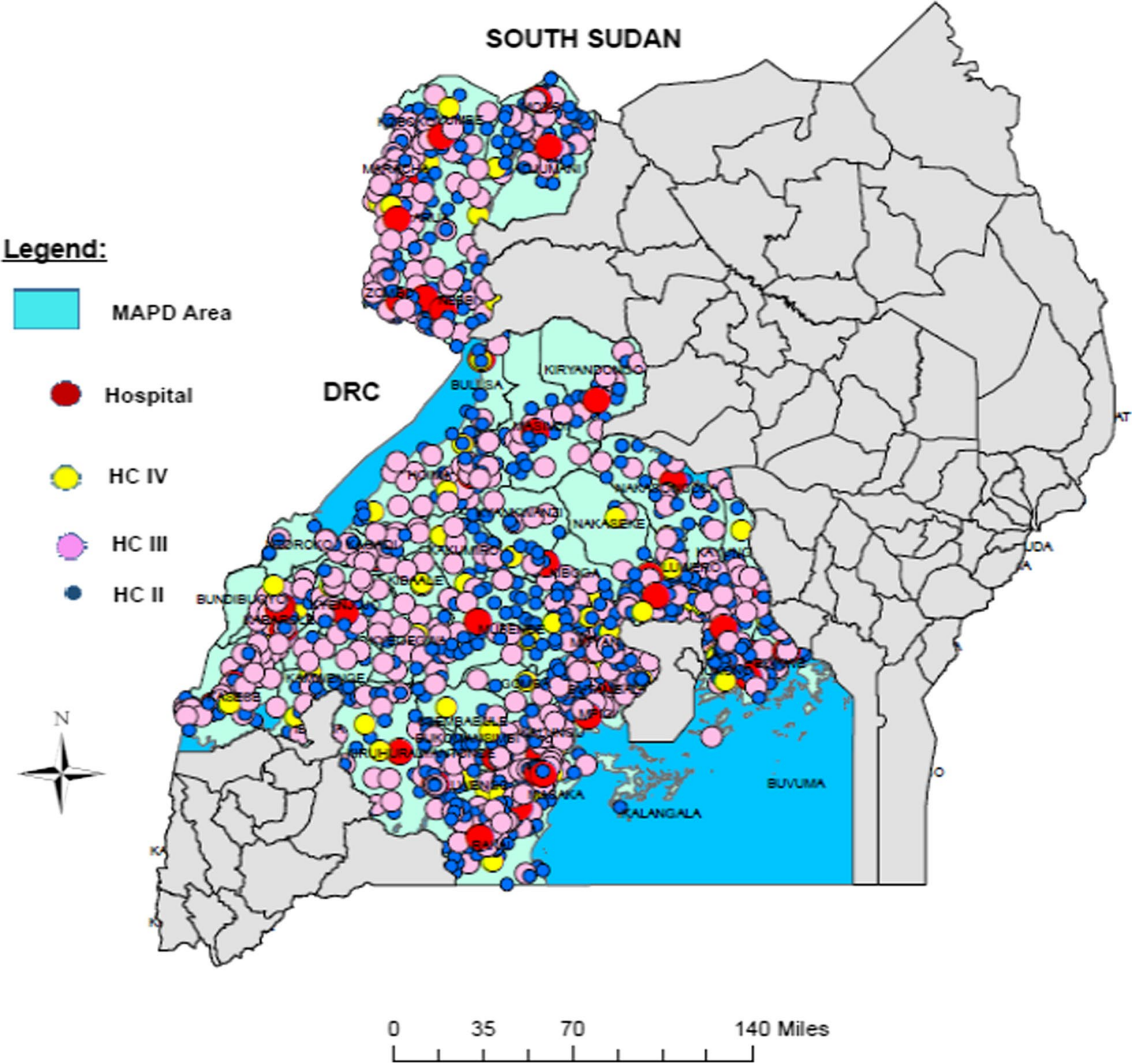
Area where health facility assessment was conducted

In addition to the routine health facility data reported into the district health information system version-2 (DHIS2), the study used data collected in a health facility survey conducted in March/April 2017 in the 52 districts in Uganda funded by the US President’s Malaria Initiative (PMI) as shown in Fig. 1. Survey data was matched using facility names and locations with the routinely collected health facility malaria data. The health facility survey followed a cross sectional design and employed quantitative research methods to assess health facility capacity to provide quality malaria care and treatment. The study collected data from all public and private health facilities in the 52 districts with the unit of analysis being the health facility. In each facility, only in-charges and heads of departments or their equivalent were interviewed. All interviewers were trained before data collection. Supervision and guidance to the field teams was provided by team supervisors who reviewed data collected each day.

### Data collection

#### Health facility survey

The Survey collected data on level of the health facility, ownership and location, availability of malaria management guidelines at the facility, malaria service provision infrastructure, supervision and malaria related training conducted at the facility as well as availability of malaria related commodities. Health facilities in Uganda are classified into levels based on the services they provide and the catchment population they intend to serve. A hospital is the highest level of Uganda’s health care structure, followed by level IV, IIIs and IIs. Level IV facilities serve a population of 100,000 people offering preventive, curative and rehabilitative care. Level III facilities are designed to serve 20,000 people offering continuous preventive, promotive and curative care services but also supervising HC IIs (serves 5000 people) under their jurisdiction [9]. Ownership of facility in Uganda is categorized as public ownership, private for profit, and private not for profit.

All facilities in Uganda are expected to have a copy of written guidelines for management of malaria. Integrated management of malaria (IMM) guidelines provides policy and information on management of fever, management of uncomplicated malaria upon confirmation and management of severe malaria including referrals from lower to higher health facilities. Availability of malaria management guidelines at the facility was measured as available or not. Similarly, health facility in-charges reported on whether or not the facility had, in their view, sufficient RDTs available to meet the expected demand in the period of the survey as well as availability of microscopy. The health facility in-charge is the health worker appointed by government to head that health facility. Supervision was measured as supervision conducted by an external authority within the last 6 months prior to the survey. The district health teams and the general hospitals are responsible for supervision of level IV health facilities. The Level IVs are responsible for supervising both the public and private HC IIIs in the district Health Center IIIs. HC IIIs are responsible for supervising HC IIs and community health workers. Supervision should be at least quarterly [10]. Training health workers in the use of RDT was measured as at least one health worker in the facility trained in the use of RDTs. In addition, the study inquired as to whether or not the facility had clocks/timers, disinfectants and power supply in the laboratory, as well as running water.

#### Routine National Health Management Information System (HMIS) data

In addition to survey data, the study used HMIS data on malaria testing and diagnosis from DHIS2 for the period March–April 2017. Data from DHIS included health facility name, number of cases suspected to have malaria. and number of suspected cases that were tested for malaria using RDTs or microscopy. The primary data collection tool for suspected malaria cases (defined as those presenting with fever) is the outpatient register. This data is collected from health facilities on a weekly basis and then entered electronically into DHIS2.

### Data management and statistical analyses

Stata 12 (StataCorp2011) software was used to merge the two data sets (survey data and DHIS2 data), clean the data and conduct subsequent analyses. Merging of the two data sets was conducted by matching district name, facility name and facility level. Of the 1620 facilities in the survey, 1174 (72%) were merged. Facilities that did not merge were mainly private for profit facilities that have not yet signed up for reporting into DHIS2.

**Fig 2:**
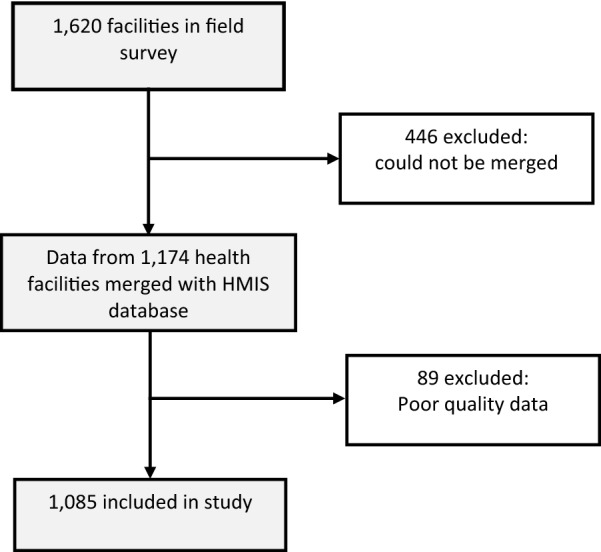
Study profile

Further cleaning after merging resulted in the removal of health facilities that had outliers (suspected malaria cases that are greater than what is the expected catchment population for the health facility by over 20%). Therefore, data from 1085 (92% of those that could be merged) facilities was analysed as shown in Fig. 2. Of these 76% (828) were public, 6% (73) were public for profit (PFPs) and 17% (184) were private not for profit (PNFP).

The outcome variable for the study was whether the facility tested more than 75% of the outpatient (OPD) attendees suspected to have malaria in the study period. The data source for suspected malaria is presence of fever as recorded in the outpatient register. The unit of analysis for the study was the health facility. Associations between the outcome and possible explanatory factors were examined using logistic regression. Factors assessed by the study included supportive supervision, availability of malaria management guidelines, clocks/timers, power supply in the laboratory, disinfectants, availability of RDTs, sharps containers, availability of running water, and training health workers in the use of RDT. First, bivariate logistic regression analyses were conducted with the outcome and each of the explanatory factors, and then factors with p-values of less than 0.2 were included in the multiple logistic regression model. Inclusion of variables with a p-value less than 0.2 allows for all pertinent and potentially influential factors to be studied [11]. The multiple logistic regression model was built with factors with a p-value of less than 0.2 resulting from the separate bivariate logistic regression models. In addition, health facility background characteristics were included.

## Results

### Background characteristics of health facilities in the study

A total of 1085 facilities were included in the final data analysis of the study. Of these, 32 were hospitals, 56 were level IV, 409 were level III, and the majority, 583, were level II, including 44 clinics/drug shops. As seen from Table 1, the majority of health facilities surveyed, 76% (828), were public facilities, 17% (184) were private not for profit (PNFP) and 7% (73) private for profit. Over 85% of the facilities surveyed were located in rural areas and over half reported to be open for service 24 h a day.

**Table 1:** Characteristics of health facilities in the study

Characteristics	N (1085)	%
Level of facility		
Hospital	32	2.9
HC IV	56	5.2
HC III	409	37.8
HC II	539	49.9
Clinic/drug shop	44	4.0
Ownership of facility		
Public	828	76.3
Private for profit	73	6.7
Private not for profit	184	16.9
Location of facility		
Rural	873	80.4
Urban	212	19.5
Open time of facility		
Open 24 h	521	48.2
Not open 24 h	560	51.8
Meeting target of malaria testing		
No	152	14.0
Yes	933	86.0
Availability of RDTs		
RDTs	996	91.8
Availability of microscopy		
Microscopy	589	54.3

### Malaria management guidelines

Analysis of survey data as seen in Table 2 shows the availability of malaria related guidelines at health facilities including integrated malaria management guidelines and malaria in pregnancy guidelines; 40% of the facilities had copies of the integrated malaria management guidelines and 38% had malaria in pregnancy guidelines. Standalone guides for management of severe malaria were, on the other hand, more available, as 65.2% of the facilities had these copies. Over 90% of the hospitals and level IV facilities had the severe malaria guidelines.

**Table 2:** Availability of guidelines for malaria diagnosis and treatment

Characteristics	N (1085)	Severe malaria treatment guidelines n (%)	National clinical guidelines n (%)	Integrated malaria management guidelines n (%)	Malaria in pregnancy guidelines n (%)	Management of uncomplicated malaria n (%)
Type of facility						
Hospital	32	28 (93.3)	21 (67.7)	20 (64.5)	25 (83.3)	29 (61.2)
HC IV	56	49 (89.1)	31 (56.3)	36 (65.4)	39 (72.2)	24 (43.6)
HC III	409	287 (70.5)	127 (31.1)	179 (44.0)	197 (48.2)	109 (26.7)
HC II	539	311 (58.2)	108 (20.2)	176 (32.9)	128 (23.9)	65 (12.2)
Clinic/drug shop	44	28 (63.6)	11 (25.0)	18 (40.9)	22 (50.0)	7 (16.1)
Ownership of facility						
Public	823	538 (64.9)	216 (26.1)	317 (38.2)	290 (35.0)	167 (20.2)
Private for profit	73	45 (61.6)	23 (31.5)	31 (42.5)	35 (48.0)	11 (15.1)
Private not for profit	184	123 (66.8)	61 (33.1)	84 (45.7)	89 (48.4)	46 (25.0)
Location of facility						
Rural	873	552 (63.2)	231 (26.5)	325 (37.2)	312 (35.7)	159 (19.4)
Urban	212	154 (72.6)	69 (32.6)	107 (50.5)	102 (48.1)	55 (26.0)
Overall	1085	706 (65.1)	300 (27.7)	432 (39.8)	414 (38.2)	224 (20.7)

### Malaria commodities

Survey data showed that most health facilities indicated malaria commodities such as RDTs and artemisinin-based combination therapy (ACT) to be available at the time of the survey, as shown in Table 3. At the time of the survey, RDTs were reported to be available at almost all (92%) facilities. Eighty-eight percent of the hospitals, 89% of level IVs, 96% of level IIIs, and 89% of level IIs reported having RDTs. Similarly, at the time of the survey, Uganda’s first line recommended treatment for uncomplicated malaria, artemether–lumefantrine (AL), and the recommended drug for the intermittent preventive treatment for malaria in pregnancy, sulfadoxine–pyrimethamine (SP) was reported to be available by most facilities, at 85% and 82%, respectively.

**Table 3:** Availability of key malaria commodities

Characteristics	N (1085)	Malaria RDTs n (%)	Artemether lumefantrine n (%)	Sulfadoxine–pyrimethamine SP n (%)
Type of facility				
Hospital	32	28 (87.5)	28 (87.5)	31 (100)
HC IV	56	50 (89.3)	51 (91.1)	54 (96.4)
HC III	409	393 (96.1)	354 (86.5)	401 (98.0)
HC II	539	482 (89.4)	444 (82.4)	496 (92.0)
Clinic/drug shop	44	40 (90.9)	38 (86.4)	41 (93.2)
Ownership of facility				
Public	828	761 (91.9)	691 (83.5)	679 (82.0)
Private for profit	73	63 (86.3)	61 (83.6)	60 (82.2)
Private not for profit	184	172 (91.8)	166 (90.2)	149 (81.0)
Location of facility				
Rural	873	807 (92.4)	732 (83.9)	718 (82.3)
Urban	212	189 (89.2)	186 (87.7)	170 (80.2)
Overall	1085	996 (91.8)	918 (84.6)	888 (81.8)

### Malaria testing at health facilities

In 2017, Uganda’s target was to have at least 75% of patients with suspected malaria tested for malaria with microscopy or a RDT. Data obtained from DHIS2 showed that 86% (933) of the facilities met this target. Private not for Profit facilities (95%) were more likely to meet the target compared to public (84%) and private for profit facilities (89%) (p-value < 0.001). There was no difference in testing rates between facilities located in rural areas (86%) compared to those located in urban areas (88%) (p-value = 0.415). Testing rates among those suspected to have malaria were also similar across facility levels [Hospital (88%), HC IV (77%), HC III (84%), HC IIs (90%), and drug shops (89%)] (p-value = 0.142).

### Factors associated with achieving target for testing suspected malaria patients

As indicated in Table 4, associations between the outcome and explanatory variables tested included: supervision in the last 6 months, availability of malaria management guidelines, availability of clocks/timers, availability of power supply in the laboratory, availability of disinfectants in the laboratory, availability of running water, availability of sharps containers, availability of RDTs, and training health workers in the use of RDT were assessed using logistic regression. From the bivariate data analyses, factors that were significantly associated with achieving the testing target (75% of the suspected malaria patients tested) included supervision in the last 6 months (OR: 2.03, 95% CI 1.26–3.28), availability of power supply in the laboratory (OR: 0.65, 95% CI 0.44–0.96), and a health facility having at least one health worker trained in the use of RDTs (OR: 1.74, 95% CI 1.14–2.64). Availability of malaria management guidelines, availability of disinfectants in the laboratory, running water, clock/timers, and sharps containers did not significantly influence meeting the malaria testing target at the health facilities.

**Table 4:** Factors associated with meeting target for malaria testing

Characteristic	Testing at least 75% of those suspected to have malaria	Crude odds ratio (95% CI)	P value	Adjusted (95% CI)	P value
Health facility type					
Public	693 (83.7)	Ref.			
Private for profit	65 (89.0)	1.58 (0.74–3.37)	0.235	1.51 (0.46–4.95)	0.500
Private not for profit	175 (95.1)	3.78 (1.89–7.58)	< 0.001	3.08 (1.49–6.38)	0.002
Location					
Rural	747 (85.6)	Ref.			
Urban	186 (87.7)	1.21 (0.76–1.89)	0.415	–	–
Level of facility					
Hospital	29 (87.5)	Ref			
HC IV	43 (76.8)	0.47 (0.14–1.59)	0.228	0.79 (0.22–2.83)	0.721
HC III	345 (84.3)	0.77 (0.26–2.27)	0.636	1.15 (0.37–356)	0.803
HC II	65 (87.9)	1.04 (0.35–3.06)	0.941	2.61 (0.82–8.25)	0.105
Clinic/drug shop	39 (88.6)	1.11 (0.27–4.52)	0.880	1.31 (0.23–7.48)	0.762
Supervision in last 6 months					
Yes	241 (91.6)	Ref.			
No	641 (84.3)	0.49 (0.30–0.79)	0.004	0.56 (0.33–0.94)	0.028
Availability of malaria management guideline					
Yes	382 (88.4)	Ref.			
No	502 (84.8)	0.91 (0.82–1.01)	0.110	0.91 (0.82–1.02)	0.120
Availability of clocks/timers					
Yes	459 (87.9)	Ref.			
No	422 (84.6)	0.75 (0.52–1.07)	0.119	0.92 (0.61–1.32)	0.676
Availability of power supply in the lab					
Yes	666 (87.6)	Ref.			
No	214 (82.3)	0.65 (0.44–0.96)	0.032	0.65 (0.40–1.04)	0.070
Availability of disinfectants in the lab					
Yes	835 (86.7)	Ref.			
No	43 (79.6)	0.59 (0.30–1.19)	0.145	0.79 (0.35–1.83)	0.588
Availability of running water					
Yes	440 (86.4)	Ref.			
No	441 (86.3)	0.98 (0.69–1.41)	0.947		
Availability of sharps containers					
Yes	859 (86.6)	Ref.			
No	23 (76.7)	0.50 (0.21–1.21)	0.126	0.56 (0.21–1.49)	0.248
Availability of RDTs					
Yes	996 (96.2)	Ref.			
No	39 (3.7)	0.69 (0.27–1.60)	0.390		
RDT training to health workers					
No HW trained	144 (80.0)	Ref.			
At least one HW trained	703 (87.4)	1.74 (1.14–2.64)	0.010	1.72 (1.09–2.71)	0.019

In the multivariate model, adjusting for level of health facility, ownership and location, predictors of a health facility achieving the testing target were supervision in last 6 months (OR: 1.72, 95% CI 1.04–2.85) and a health facility having at least one health worker trained in the use of RDTs (OR: 1.62, 95% CI 1.04–2.55).

## Discussion

In order to effectively control malaria, the WHO recommends parasitological testing of all patients suspected to have malaria before treatment [4]. In 2017, Uganda adopted national guidelines that require at least 75% of patients with suspected malaria to be tested [8] before treatment with anti-malarials. Achieving such a target coupled with adherence to test results is critical for progress in malaria control, with benefits of improving patient management, in addition to saving on the cost of ACT due to rational use. Of equal importance is collecting and recording appropriate data to allow measurement of progress on implementation of this policy. Six years ago, during the revision of HMIS tools, Uganda introduced into the outpatient register a data element for recording the presence or absence of fever, on top of patient diagnosis and treatment. Since this introduction, unpublished health facility support supervision reports have reported improvements in the completion of this data element in outpatient register, resulting in a more accurate estimation of testing rates.

This study showed high malaria testing rates among those suspected to have malaria at health facilities. Results showed that 86% of the facilities were testing over 75% of the patients suspected to have malaria; an impressive improvement from 59% in 2014 [8]. It also showed an impressive testing rate (88%) among private for-profit facilities which was slightly higher than that observed among public facilities (84%). Similarly, the Uganda malaria indicator survey 2018/19 (population-based survey) indicated that the children under 5 years of age with a fever in the 2 weeks preceding the survey who had blood taken from a finger or heel for testing could go as high as 70% in some district [12]. While adherence to test results was not the focus of this study, in Uganda adherence to test results has improved. A qualitative study conducted in rural health facilities in western Uganda in 2016 observed 55 patients, 38 tested negative and only one of these was prescribed an anti-malarial [13].

Malaria commodities were widely available in the study facilities at the time of the survey, with over 80% of the facilities reporting availability of RDTs or microscopy, ACT as well as SP. Wide availability of malaria commodities may be attributed to increased commitments, investments, and funding towards malaria control efforts by key international funding agencies such as the PMI, the Global Fund, and the UK Department for International Development (DFID) among others. For example, PMI and Global Fund resources combined increased from USD 50 million in 2008 to 219 million in 2017/18 [14, 15]. Long term availability of such commodities is, however, critical for effective malaria control.

The study showed supportive supervision and having at least one health worker trained in malaria diagnosis using RDTs were statistically significant factors in having at least 75% of patients suspected to have malaria tested. Previous studies have also demonstrated the role of supportive supervision in provision of quality health care services. One study observed noticeable improvements in maternal and newborn services following regular conduct of support supervision in 28 facilities in central Uganda [16]. Similarly, another study noted maintenance of supervision and training among features for successful interventions towards improvement of malaria related services among health care providers in sub-Saharan Africa [17]. In India, interventions combined with supportive supervision have been observed to result in better improvement in malaria control than those with no supportive supervision [18]. Study results are also consistent with what was observed in a multi-site study in Uganda where malaria testing rates at lower level facilities increased to over 90% as a result of regular support visits to health facilities [19]. Unfortunately, supportive supervision to health facilities is not always regularly provided in many developing countries for varying reasons [19]. Some studies noted that district health management teams schedule to have regular support visits, but these do not frequently happen as planned due to conflicting responsibilities on supervisors’ time and challenges with accessibility and adequacy of funds, with remote facilities most affected [20, 21]. Amidst such challenges, however, it is important for malaria control programmes to proactively support and ensure regular supervision visits to health facilities.

This study also showed that training in the use of RDT was a key determinant of high testing rates at health facilities, independent of supportive supervision. Having at least one health worker trained in the use of RDTs influenced a facility’s ability to achieve the testing target. These results correlate with other findings in Uganda, which reported very high levels of malaria testing rates following training in use of RDTs and malaria supportive supervision at health facilities [19]. While microscopy has been considered the gold standard for malaria diagnosis, its requirements (technical expertise, a functional microscope, electricity, and specialized reagents) for functionality often fall short at health facilities and where it is functional, time and human resource constraints may prevent testing of all those suspected to have malaria when patient volume is high [22]. Having at least one health worker trained in use of RDTs enables the facility to use RDTs for malaria diagnosis where and when microcopy is not functional or feasible, and/or when the patient load is high. High patient load is often the case at health facilities in Uganda, and sub-Saharan Africa in general, especially during peak malaria season [23]. Moreover, microscopic capability is often limited to high level or larger health facilities [24]. Although the presence of power supply seems to be associated with lower testing in the bivariate analysis, since association loses its significance in the multiple logistic regression model.

This study did not find availability of malaria management guidelines, availability of clocks/timers, availability of disinfectants in the laboratory, availability of running water, and availability of sharps containers significantly affecting malaria testing at health facilities. However, literature on these factors in regard to malaria diagnostic testing is also limited.

## Limitation of the study

This study used routine data reported through DHIS2. In Uganda, improvements in the quality of data reported through DHIS2 have been documented. A study assessing completeness and timeliness of these data showed significant improvements with completeness increasing from36% in 2011/12 to 85.3% in 2012/13 and timeliness of OPD data increasing from 22% (2011/12) to 78% (2012/13) [25]. Another study that assessed DHIS2 data for the period 2015–2019 for all districts in Uganda, showed that completeness of reporting by facilities was near 100% and that study noted that extreme outliers (defined as at least 3.5 standard deviations from expected value) in the data assessed were rare. In addition, the study observed data consistencies over time. Despite these improvements, however, challenges still remain including limited access to computers and internet, inadequate technical support, and limited human resources [26].

Additionally, data quality issues including inaccuracies in reporting and recording by health facilities, and transparent documentation of data corrections or adjustments by data officers, among others, still persist [27]. Nonetheless, this data still provides valuable insights into health worker practices and other health related needs. Results from DHIS2 data in this study are consistent with other studies conducted in Uganda and they therefore seem to provide a near accurate representation of what is happening in the health facilities [12].

This study did not assess individual level health worker factors that might influence malaria parasitological testing among suspected cases at health facilities. The study only looked at health facility contextual factors. As such, factors like health worker level of education, their cadre, experience, and practice of identifying patients suspected of having malaria, and other potential covariates like workload at the facility, transmission setting, health facility staffing, among others were not assessed were not examined.

## Conclusion

This study assessed predictors of high malaria testing rates in order to better understand how to support and sustain the implementation of the Uganda test and treat guideline. Results showed that supportive supervision to a health facility within 6 months and a facility having at least one health worker trained in the use of RDTs are key factors that influence a facility’s ability to achieve the target of testing at least 75% of patients with suspected malaria who report to the facility. This study underpins the value of malaria control programmes and district health teams conducting regular supportive supervision to health facilities as well as training health workers to use RDTs to ensure testing of malaria suspects before treatment.

## Data Availability

The datasets used and analysed during the current study are available from the corresponding author on reasonable request and with permission from the Health Information Department, Uganda Ministry of Health.
